# Radioclinical Profile of Eosinophilic Lung: A Case Series

**DOI:** 10.7759/cureus.62579

**Published:** 2024-06-18

**Authors:** Abir Bouhamdi, Fatima Saddouki, Badreddine Alami, Mounia Serraj, Mohamed Biaz, Mohamed Chakib Benjelloun, Bouchra Amara

**Affiliations:** 1 Pneumology Department, Hassan II University Hospital, Sidi Mohamed Ben Abdellah University, Fez, MAR; 2 Radiology Department, Hassan II University Hospital, Sidi Mohamed Ben Abdellah University, Fez, MAR

**Keywords:** asthma, churg strauss syndrome, systemic corticosteroid therapy, alveolar eosinophilia, eosinophilic lung

## Abstract

In this study, we present findings from an analysis of 17 patients diagnosed with eosinophilic lung disease, with a majority (64.70%) being male. The average age of the patients was 54 ± 13.22 years. A history of uncontrolled asthma was noted in nine cases. The clinical picture was characterized by persistent dyspnea and cough. Blood hypereosinophilia was present in all cases, with a median of 1770 cells/ul. Two patients had a pulmonary eosinophilia greater than 25%. Radiological findings were consistent with diffuse bilateral ground-glass opacities or areas of consolidation in the majority of cases. The main etiologies identified were chronic eosinophilic pneumonia (12 cases), followed by eosinophilic granulomatosis with polyangiitis (3 cases), idiopathic hypereosinophilic syndrome (1 case) and drug-induced hypereosinophilia (1 case). All patients were treated with systemic corticosteroids, with the addition of immunosuppressive therapy necessary in three cases. Notably, five relapses were recorded after corticosteroid therapy was stopped.

## Introduction

Eosinophilic lung diseases are a complex group of diffuse parenchymal lung diseases characterized by eosinophilic infiltration of lung tissue [1]. Although rare, these conditions are of significant clinical importance due to their diagnostic and therapeutic implications. Eosinophils, immune cells involved in allergic and inflammatory responses, are often found in excessive quantities in lung tissue, associated with alveolar eosinophilia exceeding 25%. However, blood eosinophilia can vary, sometimes being absent or temporary [2].

These conditions share similarities in pathophysiology, radiology, and response to corticosteroid treatment, but they differ considerably in terms of etiology, clinical presentation, and disease progression. The underlying mechanisms involved in these pathologies often remain complex and poorly understood, making their diagnosis and clinical management a challenge for practitioners.

Eosinophilic lung diseases can be classified into primary and secondary categories [1]. Primary eosinophilic lung diseases originate directly from eosinophilic infiltration of the lung tissue, often without a clear underlying cause. Examples include eosinophilic pneumonia and eosinophilic granulomatosis with polyangiitis (formerly known as Churg-Strauss syndrome). Secondary eosinophilic lung diseases arise due to underlying conditions or triggers such as parasitic infections, allergic reactions, certain medications, or other systemic diseases. These include conditions like allergic bronchopulmonary aspergillosis and hypereosinophilic syndrome. Primary forms typically have a more direct association with eosinophilic infiltration, while secondary forms are often linked to external factors or underlying diseases.

Our research aims to thoroughly investigate the epidemiological, clinical, biological, and radiological characteristics of patients with eosinophilic lung disease. By emphasizing the varied and often insidious clinical presentations of these conditions, as well as the diagnostic challenges faced by clinicians, our study aspires to contribute to improving the management of patients with eosinophilic lung disease. By providing relevant data on the different subtypes of this pathology and analyzing responses to available treatments, we aim to contribute to a better understanding of these conditions and guide therapeutic decisions in daily clinical practice.

## Materials and methods

Study design

This retrospective study was conducted at the Hassan II University Hospital in Fez, Morocco, over a six-year period from January 1, 2017, to March 7, 2023. The aim was to analyze the epidemiological, clinical, biological, and radiological characteristics of patients presenting with eosinophilic pneumonitis.

Study population

The study population included all patients whose medical records indicated a confirmed diagnosis of eosinophilic lung disease, with eosinophilic infiltration of lung tissue and/or alveolar eosinophilia and/or blood eosinophilia. Inclusion criteria were broad to include all possible cases of eosinophilic pneumonitis. The inclusion criteria were as follows: a clinical diagnosis of eosinophilic lung disease confirmed by radiological and biological findings, alveolar eosinophilia, and eosinophilia in blood or induced sputum. Patients of all ages and both sexes were included, provided that a complete medical record was available for data extraction.

Data collection

Epidemiological data (age, sex), medical history (including the presence of asthma, other allergies, recent onset of smoking for acute eosinophilic pneumonia (AEP), drugs for drug-induced eosinophilic pneumonia, malignancy, radiation exposure), respiratory symptoms, results of biological examinations (blood eosinophil levels), results of radiological examinations (including chest scans), results of respiratory function tests, treatments received, and clinical course were extracted from patients' medical records.

Data analysis

A descriptive analysis was performed to describe the demographic, clinical, biological, and radiological characteristics of the patients. Quantitative variables were expressed as mean ± standard deviation and qualitative variables as percentages. The data were compared with the results of other studies in the discussion section.

Validation of results

Results were validated by comparing data extracted from medical records with established diagnostic criteria for eosinophilic lung.

## Results

Description of the population

Seventeen cases were studied, comprising 11 men and 6 women, with a male-to-female ratio of approximately 1.83:1. The mean age at diagnosis was 54 ± 13.22 years, ranging from 20 to 72 years. The mean age at onset of symptoms was 47.11 years. Seven patients were former smokers who had quit years ago, while two were still smoking.

Seven patients had a history of rhinitis and allergic conjunctivitis, while nine had a history of asthma. Six patients had uncontrolled late-onset asthma, with a mean age of onset of 49.83 years, while three had uncontrolled early-onset asthma, with a mean age of onset of 29.33 years.

Clinically, all patients had dyspnea for months or even years prior to treatment. Eight cases had stage II dyspnea according to the modified Medical Research Council (mMRC) scale, while six cases were classified as mMRC stage III. Three patients were admitted in respiratory distress requiring an initial stay in intensive care. Sixty-four percent of patients had a dry cough, while 35% had a productive cough. Three patients reported episodes of low-grade hemoptysis and four cases complained of atypical chest pain. Six cases had inflammatory polyarthralgias, seven had skin urticaria, and four had neurological symptoms such as limb paresthesias and dizziness. Three patients complained of abdominal pain. On pulmonary examination, crepitating rales were found in 11 patients and sibilant rales in 3 others.

Biologically, the median level of circulating eosinophils was 1770 cells/µL. Among the etiological tests, total IgE was elevated in three cases with a mean of 243.27 ± 93.95 IU/ml, while specific IgE antibody titers against *Aspergillus *were negative in all patients. Cytobacteriological examination of the urine revealed microscopic hematuria in seven cases and positive 24-hour proteinuria in four cases. Anti-neutrophil cytoplasmic antibodies (ANCA) were positive in only one of the 17 cases. Stool parasitology was performed in all cases, revealing the presence of *Entamoeba histolytica* in two.

**Figure 1:**
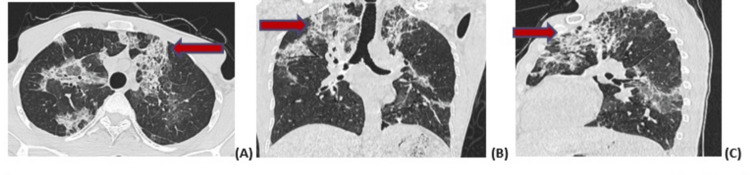
Figure 1 (A-C): CT scans in axial, coronal, and sagittal sections showing crazy paving (red arrow) extending to both lung fields, which can be indicative of eosinophilic lung disease among other conditions

**Figure 2:**
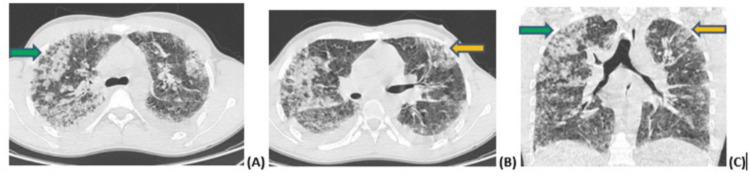
Figure 2 (A-C): CT scan in axial and coronal sections showing foci of condensation (green arrow) and ground-glass areas (yellow arrow), predominantly apical

**Figure 3:**
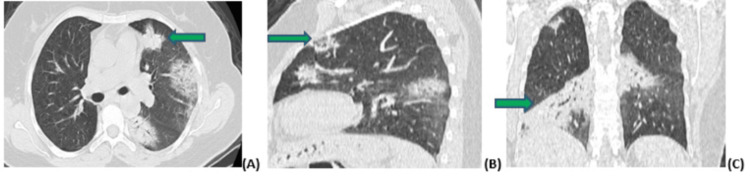
Figure 3 (A-C): Axial, sagittal, and coronal CT sections showing foci of pulmonary condensation marked at the level of the left superior lobe (green arrow), forming part of chronic eosinophilic pneumonia

On chest CT, all but one of the patients had diffuse bilateral pneumonitis with a mixed (central and peripheral) pattern. Radiological findings included air space consolidation predominating in the peripheral regions, especially in the upper lobes, typical of chronic eosinophilic pneumonia. Additionally, diffuse ground-glass opacities and interlobular septal thickening were observed in cases of AEP (Figures 1-3). Bronchoalveolar lavage (BAL) was performed in 11 cases, revealing eosinophilia greater than 25% in 2 cases and ranging from 4% to 20% in the others. Eosinophils were detected in induced sputum in five cases.

An osteomedullary biopsy was performed in one case because the patient had a very high eosinophil count of 20,940 cells/mm³, raising suspicion of a myeloproliferative or lymphoproliferative hypereosinophilic syndrome. The biopsy showed marked bone marrow eosinophilic infiltration.

Transthoracic echocardiography was performed in all cases to assess the impact of blood hypereosinophilia, revealing various cardiac pathologies in three patients. Electromyography was also conducted in all cases, with results showing no abnormalities.

Respiratory function tests revealed restrictive ventilatory disorders in six patients, obstructive in five, and mixed in five others. Total lung capacity (TLC) was low in three patients, with a mean of 48.66%. The six-minute walk test revealed exercise-induced desaturation in two patients. One patient did not undergo pulmonary function testing due to severe respiratory distress.

Twelve patients were diagnosed with chronic eosinophilic pneumonia after a thorough etiological work-up revealed no specific cause. Eosinophilic granulomatosis with polyangiitis (EGPA), involving pulmonary, articular, neurological, and renal systems, was observed in three patients. Additionally, a case of drug-induced pneumonia was identified in a young patient, associated with the use of Triaxon (Ceftriaxone), and another patient was diagnosed with idiopathic hypereosinophilic syndrome.

Once the diagnosis of eosinophilic lung had been established, systemic corticosteroid therapy was prescribed in all patients, combined with immunosuppressive therapy in three cases of vasculitis. One patient received bolus cyclophosphamide followed by a transition to azathioprine, while two others were treated with anti-TNF alpha (rituximab). Avoidance of the offending drug was sufficient to normalize blood hypereosinophilia levels in one patient (Table 1).

**Table 1:** Descriptive analysis of the study population: study of 17 cases BAL: bronchoalveolar lavage; ANCA: anti-neutrophil cytoplasmic antibodies; PFT: pulmonary function test

Patient	1	2	3	4	5	6	7	8	9	10	11	12	13	14	15	16	17
Sex	M	F	M	M	M	F	F	F	M	M	M	M	M	M	F	M	F
Age	64	57	56	52	79	40	46	52	52	65	60	45	51	20	72	59	48
Age of onset of symptoms	59	54	24	50	73	40	46	31	52	64	40	43	48	20	63	48	46
Smoking	Yes	No	Yes	No	Yes	No	No	No	Yes	Yes	Yes	No	Yes	Yes	No	Yes	No
Allergic rhinitis	Yes	Yes	No	No	Yes	No	No	No	No	No	Yes	Yes	Yes	No	No	No	Yes
Asthma	Yes	No	Yes	No	No	Yes	Yes	Yes	No	No	Yes	No	No	No	Yes	Yes	Yes
Dry cough	Yes	Yes	Yes	Yes	No	Yes	Yes	No	Yes	No	No	Yes	No	Yes	No	Yes	Yes
Sputum	No	No	No	No	Yes	No	No	Yes	No	Yes	Yes	No	Yes	No	Yes	No	No
Dyspnea	Yes	Yes	Yes	Yes	Yes	Yes	Yes	Yes	Yes	Yes	Yes	Yes	Yes	Yes	Yes	Yes	Yes
Chest pain	No	No	Yes	No	No	No	No	No	Yes	No	No	Yes	No	No	Yes	No	No
Joint signs	Yes	Yes	No	No	No	No	Yes	No	No	No	No	No	No	No	Yes	Yes	Yes
Skin signs	Yes	No	No	No	No	No	Yes	Yes	No	Yes	Yes	No	No	No	No	Yes	Yes
Digestive signs	No	No	No	No	No	No	No	Yes	No	No	Yes	No	Yes	Yes	No	No	Yes
Neurological signs	No	No	No	No	No	No	No	No	No	No	No	Yes	No	No	Yes	Yes	Yes
Crepitating rales	Yes	Yes	Yes	No	No	Yes	Yes	No	Yes	Yes	No	Yes	No	Yes	Yes	No	Yes
Wheezing	No	No	No	Yes	No	No	No	No	No	No	No	No	Yes	No	No	Yes	No
Foci of consolidation	Yes	Yes	Yes	No	No	Yes	No	-	Yes	Yes	No	Yes	No	Yes	Yes	Yes	Yes
Ground-glass opacities	Yes	No	Yes	Yes	Yes	Yes	Yes	-	Yes	No	Yes	No	Yes	Yes	No	No	No
Blood eosinophil count (cells/mm^3^)	1430	2070	2160	2110	1823	710	650	1070	1600	1770	681	8896	20940	2100	3530	1630	900
Sputum eosinophil count	-	-	-	-	6%	-	-	5%	-	-	-	-	6%	-	-	5%	4%
Eosinophil count in BAL	10%	Not made	26%	12%	Not made	12%	20%	16%	53%	10%	4%	12%	Not made	Not made	10%	Not made	Not made
Hematuria	No	No	Yes	No	No	No	Yes	No	No	Yes	No	Yes	No	No	Yes	Yes	Yes
ANCA	Negative	Negative	Negative	Negative	Negative	Negative	Negative	Negative	Negative	Negative	Negative	Negative	Negative	Negative	Negative	Positive	Negative
Stool parasitology	Negative	Entamoeba histolitica	Negative	Negative	Entamoeba histolitica	Negative	Negative	Negative	Negative	Negative	Negative	Negative	Negative	Negative	Negative	Negative	Negative
PFT pattern	Mixed ventilatory disorder	Obstructive ventilatory disorder	Restrictive ventilatory disorder	Obstructive ventilatory disorder	Obstructive ventilatory disorder	Restrictive ventilatory disorder	Restrictive ventilatory disorder	Obstructive ventilatory disorder	Normal	Mixed ventilatory disorder	Mixed ventilatory disorder	Restrictive ventilatory disorder	Mixed ventilatory disorder	-	Obstructive ventilatory disorder	Mixed ventilatory disorder	Restrictive ventilatory disorder
Diagnosis retained	Chronic eosinophilia pneumonia	Chronic eosinophilia pneumonia	Chronic eosinophilia pneumonia	Chronic eosinophilia pneumonia	Chronic eosinophilia pneumonia	Chronic eosinophilia pneumonia	Chronic eosinophilia pneumonia	Chronic eosinophilia pneumonia	Chronic eosinophilia pneumonia	Chronic eosinophilia pneumonia	Chronic eosinophilia pneumonia	Chronic eosinophilia pneumonia	Hypereosinophilic syndrome	Acute drug-induced pneumonia	Eosinophilic granulomatosis with polyangiitis (EGPA)	Eosinophilic granulomatosis with polyangiitis (EGPA)	Eosinophilic granulomatosis with polyangiitis (EGPA)
Corticosteroid therapy	Yes	Yes	Yes	Yes	Yes	Yes	Yes	Yes	Yes	Yes	Yes	Yes	Yes	Yes	Yes	Yes	Yes
Immunosuppressive treatment	No	No	No	No	No	No	No	No	No	No	No	No	No	No	Yes	Yes	Yes

 

 

 

 

## Discussion

Eosinophilic lung diseases represent a rare and heterogeneous group of diffuse parenchymal lung diseases. The systematic search for an underlying cause, whether parasitic, fungal, or drug-induced, is essential for the practitioner. In the absence of an identified cause, the diagnostic spectrum encompasses acute or chronic eosinophilic pneumonia, EGPA, or hypereosinophilic syndromes, whether lymphocytic, myeloproliferative, or idiopathic.

In our series, the mean age observed was 47 years, in line with data reported in various medical studies, such as the series by Achrane et al. [3] with a mean age of 43 years, and that by Msika [4] with a mean age of 45 years. We noted 9 cases with a history of asthma, compared with 6 cases in Aouadi's series [5], 16 cases in Achrane's [3], and 6 cases in Raftani's [6]. The predominant clinical symptoms were cough and dyspnea, consistent with previous observations [3-6]. Etiologies included 12 cases of chronic eosinophilic pneumonia, 3 cases of EGPA (formerly known as Churg-Strauss syndrome), 1 case of idiopathic hypereosinophilic syndrome, and 1 case of drug-induced AEP. Chronic eosinophilic pneumonia, also known as Carrington's disease, was frequently seen, with 12 cases documented in our study. Although this disease can occur at any age, the majority of patients were diagnosed between the ages of 30 and 50, with a mean age of 54.16 years in our series. Despite previous reports indicating a female predominance (64%) in Msika's series [4]), our series presented a male predominance with a sex ratio of two.

A significant correlation between chronic eosinophilic pneumonia and allergic diseases has been demonstrated, with more than half of patients presenting with allergic disorders such as bronchial asthma, atopic dermatitis, and allergic rhinitis [7]. In our series, nine patients were asthmatic and a further seven had symptoms of allergic rhinitis.

Blood eosinophilia, accompanied by elevated IgE levels, was observed in the majority of patients with chronic eosinophilic pneumonia. In our cohort, circulating hypereosinophilia was present in 11 chronic eosinophilic pneumonia patients, with a mean of 1882.25 cells/mm³. Bronchoscopic examinations often revealed eosinophilia in BAL and/or transbronchial lung biopsies (TBLB). Radiological abnormalities typical of chronic eosinophilic pneumonia included foci of consolidation and ground-glass opacities, mainly localized in peripheral areas of the lungs, particularly in the upper and middle regions. Patients with chronic eosinophilic pneumonia generally responded favorably to corticosteroids, as demonstrated by Naughton et al. [8] in a recent series of 12 cases with a mean follow-up of 10.2 years, although the spontaneous resolution was rare (less than 10%). Treatment with corticosteroids usually begins with 1 mg/kg prednisolone, with progressive tapering over a period of 6 to 12 months, although no optimal treatment regimen has been established. Relapses were frequent, occurring in over half of all chronic eosinophilic pneumonia cases.

Numerous drugs and toxic substances have been identified as potential triggers for acute or chronic eosinophilic pneumonitis [9-10]. Diagnosis of these drug-induced conditions was often complex, as they could manifest at variable times after drug administration and regress rapidly after drug discontinuation. The concomitant occurrence of skin rash, pleural effusion, and radiological infiltrates strongly suggested a drug-induced origin. In our series, one case of cutaneous rash occurred on the fifth day of treatment with third-generation cephalosporins. In addition, eosinophilia greater than 25% in the BAL was considered a diagnostic clue. Primary treatment consisted of discontinuing the offending drug or toxic substance. In severe, acute cases, corticosteroids could be considered.

Hypereosinophilic syndrome is a rare disorder, generally presenting between the ages of 20 and 50, and predominantly in men. Diagnosis is based on criteria established by Chusid in 1975 [11], including persistent elevation of the eosinophil count in the blood, greater than or equal to 1500 cells per mm³ for more than six months, associated with visceral clinical signs such as hepatosplenomegaly or cardiac, neurological, pulmonary, or cutaneous involvement, and excluding any other known cause of hypereosinophilia. Three subtypes of hypereosinophilic syndrome are distinguished: lymphoproliferative, myeloproliferative, and idiopathic. Cardiac involvement, found in 58% of patients, has a major impact on vital prognosis. Given the favorable response to corticosteroid therapy in around half of all patients, initial treatment with corticosteroids is often recommended, starting at a dose of 1 mg/kg/day for six weeks and tapering off over six months. Relapses are frequent.

EGPA, formerly known as Churg-Strauss syndrome, is characterized by necrotizing vasculitis of small vessels [12]. According to the criteria of the American College of Rheumatology [13], its diagnosis is based on the presence of at least four of the following six criteria: asthma, hypereosinophilia, sinusitis, pulmonary infiltrate, histological evidence of vasculitis, and polyneuritis. Although it can occur at any age, EGPA mainly affects individuals between the ages of 40 and 50, affecting men and women equally. BAL analyses often reveal a high eosinophilia, sometimes over 60%. ANCA, particularly of the pANCA type directed against neutrophil myeloperoxidase (MPO), are present in around two-thirds of patients [14]. The prognosis of EGPA has improved markedly with the use of systemic corticosteroids and immunosuppressants, such as cyclophosphamide and azathioprine, in resistant cases. The choice of treatment is guided by the severity of the disease, assessed by a Five Factor Score (FFS) [15]. All three patients described in our study had a favorable outcome (Table 2).

**Table 2:** Percentage frequency of clinical manifestations in nine Churg Strauss syndrome series

Authors	Guillevin (1987) [16]	Gaskin (1991) [17]	Guillevin (1999) [18]	Solans (2001) [19]	Della Rossa (2002) [20]	Keogh (2003) [21]	Sinico (2005) [22]	Our series (2023)
Numbers	43	21	96	32	19	91	93	3
Gender (M/F)	23/19	14/7	52/44	9/23	9/10	51/40	39/54	½
Age (average)	43.2 (7-66)	46.5 (23-69)	48 (17-74)	42.5 (17-85)	46.3 (25-67)	49.1 (10-77)	51.6 (18-86)	56 (48-72)
Asthma	100	100	100	100	100	99	96	12
General signs	72	-	68	69	79	-	68	-
Lung infiltrates	77	43	37	53	37	58	50	6
Allergic rhinitis	21	-	-	62	-	-	-	6
Multineuritis	67	70	78	66	58	75	64	12
Digestive disorders	37	58	33	37	47	31	21	6
Heart disease	49	15	36	28	31	13	16	6
Arthralgia, polyarthritis	28	43	40	37	-	30	-	12
Cutaneous signs	50	-	51	69	-	57	53	6
Kidney damage	16	81	26	12	21	25	27	18
Pleuresis	-	-	-	3	10	-	-	-

We acknowledge two important limitations of our study. First, due to its retrospective nature, we were confronted with missing or incomplete data, which may have influenced certain aspects of our analysis. Second, the limited duration of the study restricted our ability to draw definitive conclusions about the long-term course of the disease. Additionally, the relatively small number of included patients may also be a limitation. These limitations must be taken into account when interpreting results and extrapolating conclusions.

## Conclusions

The clinical and etiological diversity of pulmonary disorders with an eosinophilic component underlines the crucial importance of a precise diagnostic and therapeutic approach. Our study, carried out on a cohort of patients with eosinophilic lung disease, highlights a variety of clinical presentations, underlying etiologies, and responses to treatment. The results reveal a predominance of pathologies such as chronic eosinophilic pneumonia, EGPA, and hypereosinophilic syndrome, each with distinct clinical features and specific therapeutic implications. Rapid and accurate identification of these often complex and potentially serious conditions is essential to guide clinical management and improve patient prognosis.

Treatment options, ranging from avoidance of triggering agents to the use of corticosteroids and targeted therapies, underline the need for an individualized approach based on a thorough understanding of each case. By providing new insights into these rare but significant lung conditions, our study aims to inform clinical practice and stimulate future research in this complex and evolving field.
